# Editorial: Screening and verification of new targets for CAR-T immunotherapy in cancer

**DOI:** 10.3389/fimmu.2023.1189773

**Published:** 2023-04-11

**Authors:** Yang Su, Chen Yuan, Ming Shi

**Affiliations:** ^1^ Cancer Institute, Xuzhou Medical University, Xuzhou, Jiangsu, China; ^2^ Center of Clinical Oncology, The Affiliated Hospital of Xuzhou Medical University, Xuzhou, Jiangsu, China; ^3^ Jiangsu Center for the Collaboration and Innovation of Cancer Biotherapy, Xuzhou Medical University, Xuzhou, Jiangsu, China; ^4^ Dana-Farber Cancer Institute, Harvard Medical School, Boston, MA, United States

**Keywords:** CAR-T, new targets, immunotherapy, prediction method, clinical efficacy

Chimeric antigen receptor T (CAR-T) cells have shown promising efficacy in treating hematological malignancies, particularly CD19 CAR-T for B-cell acute lymphoblastic leukemia with a 70~94% complete remission rate (1). However, antigen escape presents a significant challenge for the long-term effectiveness of CAR-T (1–3), and using CAR-T to treat solid tumors faces obstacles due to the lack of safe and effective treatment targets (1). Therefore, finding new targets for CAR-T therapy is critical.

The ideal target of CAR-T therapy should be specifically expressed or remarkably up-regulated on the surface of tumor cells. In addition to this classic target screening method, there are also some new screening methods that deserve attention. For example, peptide-centric CARs have the potential to vastly expand the pool of immunotherapeutic targets to include non-immunogenic intracellular oncoproteins (4). In addition to screening new targets on tumor cells, we can also focus on targets on CAR-T cells, such as canonical BRG1/BRM-associated factor (5) and PD1 (6). The strategy of obtaining potential targets for immunotherapy through high-throughput data analysis (Chen et al.) has crucial implications for screening new targets for CAR-T therapy. The use of multidimensional omics data advanced CAR-T cell therapy (7). DNA sequencing have identified numerous tumor-associated somatic mutations, some of which might generate tumor-specific neoantigens and could potentially serve as novel targets for CAR-T therapy (8, 9). Genome-wide pooled CRISPR–Cas9 knockout library screening has resulted in the identification of key genes involved in T cell cytotoxicity (10) and genetic alterations in tumor cells that influence resistance to treatment (11). Epigenetic reprogramming of CAR-T cells also has the potential to enhance T cell cytotoxicity (12). Meanwhile, integrating proteomics and transcriptomics is also a reliable strategy for screening CAR-T therapeutic targets (13).

Several new targets for CAR-T therapy in hematological malignancies have been reported. GPRC5D has been identified as a potential target for CAR-T treatment of multiple myeloma in preclinical research by Smith et al. (14) and confirmed by subsequent clinical trials (15–17). In this Research Topic, Wu et al. suggested in a preclinical study that CD70 is a potential target for CAR-T treatment of acute myeloid leukemia (Wu et al. ). Additionally, B7-H3 CAR-T cells have been shown to have potent preclinical activity against pediatric solid tumors and brain tumors (18).

In addition to discovering a single new target, the combined application strategy of multiple targets has also become an important development direction for CAR-T therapy. CAR-T sequential therapy targeting BCMA and CD19 has achieved promising clinical results (19). Perna et al. showed that the combined targeting strategy of ADGRE2, CCR1, CD70, and LILIB2 can effectively improve patient coverage of CAR-T treatment (13).

Although new targets are constantly being discovered, there is still a need of effective prediction methods for their clinical efficacy. Several researches have constructed prognostic models of CD19 CAR-T therapy in B cell acute lymphoblastic leukemia (11, 20) or large B cell lymphoma (21–23) based on clinical information and high-throughput data (24). PET/CT was used to predict prognosis of B-cell lymphoma treated with CD19/CD22 dual-targeted CAR-T (25). In this Research Topic, several studies have also successfully predicted the immunotherapy and prognosis of different cancers, including hepatocellular carcinoma (Liu et al.), early-onset gastric cancer (Liu et al.), lung cancer (Zhang et al.), and gliomas (Han et al.), based on clinical information and high-throughput data of patients. These prediction models have significant reference values for predicting the efficacy of CAR-T treatment in solid tumors.

CAR-T cells provide remarkable opportunities and are expected to become an effective means of treating multiple tumors in the future. However, current challenges include antigen escape relapse resulting from selective immune pressure of CAR-T cells and the lack of ideal targets in solid tumors. Moreover, the lack of clinical efficacy prediction means of CAR-T therapy also hinders its application and promotion to some extent. Our Research Topic provides valuable references for screening new targets of CAR-T and predicting its efficacy. We invite you to read each of these enlightening articles.

## Author contributions

All authors have made a substantial, direct, and intellectual contribution to the work and approved it for publication.
